# Upfront Thoracic Magnetic Resonance Imaging for the Evaluation of Thymic Lesions to Reduce Non-Therapeutic Diagnostic Thymectomy: A Narrative Review

**DOI:** 10.3390/healthcare12202036

**Published:** 2024-10-14

**Authors:** Khang Duy Ricky Le, Annie Jiao Wang, Shasha Haycock, Kaylah Fink, Su Jin Lee

**Affiliations:** 1 Department of Surgery, Northeast Health Wangaratta, Wangaratta, VIC 3677, Australia; 2 Department of General Surgical Specialties, The Royal Melbourne Hospital, Melbourne, VIC 3052, Australia; 3 Geelong Clinical School, Deakin University, Geelong, VIC 3220, Australia; 4 Faculty of Medicine, Dentistry & Health Sciences, The University of Melbourne, Melbourne, VIC 3052, Australia; 5 Department of Radiology, The Royal Melbourne Hospital, Melbourne, VIC 3052, Australia

**Keywords:** magnetic resonance imaging, computed tomography, thymic lesion, thymoma, thymic cyst, thoracic imaging, thoracic surgery

## Abstract

**Background:** Thymic pathologies represent the most common lesions of the anterior mediastinum. They may be classified as malignant or benign. Current diagnostic pathways recommend an initial assessment with computed tomography (CT) imaging to delineate potentially malignant thymic lesions. Despite this, high rates of non-therapeutic thymectomy continue to be observed. This carries with it significant anaesthetic, operative, and post-operative risks, in addition to healthcare costs. Consequently, there is a growing interest in magnetic resonance imaging (MRI) as a primary diagnostic modality for lesions of the anterior mediastinum. This narrative review outlines the current approaches to the evaluation of thymic lesions, with a discussion of the strengths and limitations of CT and MRI imaging modalities. It also evaluates the current discourse on the use of upfront MRI for thymic and anterior mediastinal lesion assessment. **Methods:** A narrative review was performed following a search on the Medline database. Articles that were evaluated had explored the role of MRI on the evaluation of thymic and anterior mediastinal lesions. **Results:** Current work-up for thymic and anterior mediastinal lesions are highly variable and centre around the use of CT. Upfront MRI demonstrates a similar accuracy to CT for various thymic and anterior mediastinal pathologies; however, the efforts to integrate this approach into routine practice remain in their infancy, with no standardised guidelines that exist. **Conclusions:** This narrative review demonstrates that there is a paucity of evidence relating to the sensitivity and specificity of MRI compared to CT for thymic lesion analysis and their subsequent relationship with non-therapeutic thymectomy. Future prospective trials to assess the role of MRI in thymic lesion determination are required to understand whether MRI can more accurately characterise these lesions to reduce non-therapeutic thymectomy. Additionally, further research efforts are required to characterise best-practice methods for integrating MRI into diagnostic pathways for these lesions in a cost-effective and resource-conscious manner.

## 1. Introduction

Anterior mediastinal lesions are uncommon, with an estimated prevalence of 0.49% to 0.89% [1]. They include a spectrum of benign conditions including dermoid cysts, thyroid cysts, thymic cysts, and other benign thymic lesions, as well as malignant conditions including thymoma and lymphoma [2,3]. Of these, lesions of the thymus represent the most common conditions encountered [1].

The approach to the thymic lesion is highly varied due to the diversity of causes and therefore it remains an area of diagnostic difficulty for clinicians. Current diagnosis is based on computed tomography (CT), which is challenged for its low sensitivity [4,5]. In addition, CT also poses additional risks from ionising radiation [4,5]. The challenges with CT are perhaps exemplified by the high rates of non-therapeutic thymectomy, which is defined as the removal of the thymus without any therapeutic benefit due to the final diagnosis representing benign and non-life-threatening conditions (such as thymic cysts and thymic hyperplasia), in the order of 22% to 69% [4,5]. This has significant impacts, including the risk of exposure to general anaesthesia and surgery without a clear benefit, in addition to healthcare costs. Furthermore, the lack of a clear diagnosis, multiple referral pathways and unnecessary surgery are likely to have significant impacts on the quality of life and mental health of patients, as well as their capacity to maintain daily commitments including work and family obligations.

Magnetic resonance imaging (MRI), which confers higher soft-tissue resolution, has the potential for improved characterisation of thymic lesions to avoid unnecessary surgical intervention [6,7]. This is backed by emerging evidence supporting the role of earlier MRI in the characterisation of these lesions with greater accuracy [2]. Despite this, the diagnostic pathways for lesions of the thymus and overall anterior mediastinal lesions remain poorly standardised, with significant heterogeneity in CT protocols. Furthermore, there are additional challenges with integrating more upfront MRI into routine guidelines due to cost, expertise, resource limitations, and practicality. This review therefore explores the role of upfront MRI in characterising anterior mediastinal lesions and offers insights into the development of more standardised diagnostic pathways for these, in order to avoid non-therapeutic thymectomy.

## 2. Current Diagnostic Approaches to the Thymic Lesion

Accurate diagnoses of anterior mediastinal pathologies remain an area of ongoing research. Currently, there are varied approaches to diagnostic work-up. This has multiple implications for patients and health systems through the delicate balance between the appropriate management and overdiagnosis and the unnecessary treatment with non-therapeutic thymectomy.

According to the European Society for Medical Oncology (ESMO), the work-up for anterior mediastinal lesions should be based on CT imaging [8]. The rationale for this is that CT is equal to or superior to MRI for the diagnosis of anterior mediastinal lesions. Despite this, there is a suggestion that CT may be inferior to MRI in identifying cystic lesions [9]. The most recent evidence-informed algorithm for thymic lesion work-up has been proposed by the British Thoracic Oncology Group (BTOG) [10]. This approach provides recommendations for a further evaluation of the thymic lesion, as well as other anterior mediastinal lesions, based on CT imaging. The algorithm sets specific standards about the quality of CT imaging to be performed. Specifically, non-contrast or arterial phase CT of the neck and chest are considered suboptimal due to their reduced sensitivity in delineating anterior mediastinal lesions [10]. For clinicians, the recommendation is contrast-enhanced delayed-phase CT of the neck and chest [10].

In cases of thymic hyperplasia identified on CT, no follow-up is recommended unless there is a diagnostic uncertainty or a need for surveillance, whereby contrast-enhanced MRI is recommended [10]. For thymic cysts, contrast-enhanced MRI is recommended for larger lesions (>2 cm) as well as for surveillance [10]. For atypical cysts and suspected early stage thymoma, consideration is given to bypassing further imaging and progressing towards thymectomy followed by MRI surveillance [10]. For advanced or aggressive malignancies, further staging to include full body positron emission tomography (PET) imaging and consideration of biopsy for tissue diagnosis is suggested [10]. It is clear from these guidelines that BTOG emphasises the importance of MRI in the later stages of the evaluation of thymic lesions; however, there remains the potential to consider expediting MRI to the point of index diagnosis, rather than using additional contrast-enhanced CT in the first instance. It is unclear at this point in time how widely accepted these recommendations are, or how effective these guidelines are in reducing non-therapeutic thymectomy. Further research is required to determine whether this approach serves to improve the judicious healthcare resource allocation; however, it is clear from the reliance on MRI modalities for work-up that upfront MRI may be more efficient.

## 3. An Overview of Radiologic Investigations for Thymic Lesions

Current imaging modalities to evaluate thymic lesions include chest radiography (CXR), CT, MRI, and PET. Additionally, the use of contrast-enhanced ultrasound (CEUS) has also been proposed. Differentiating between normal and pathological thymus may be difficult; however, a reliable diagnostic pathway would be advantageous in avoiding unnecessary biopsy or surgery. Furthermore, a more efficient and expedited diagnosis would also provide patients with more peace of mind and less uncertainty during the diagnostic process. Imaging also plays a vital role in the staging and stratification of patients for therapy and in establishing a prognosis. Herein, we review the various radiologic modalities for thymic lesion assessment with a focus on the important thymic pathologies. We first cover the role of CXR, followed by an evaluation of benign, pre-malignant and malignant lesions of the thymus and anterior mediastinum on CT and MRI. We then conclude the section by discussing additional modalities including CEUS and endobronchial ultrasound.

### 3.1. Chest Radiography

Chest radiography is a quick, inexpensive, and low-radiation initial imaging modality, which may suggest or incidentally detect thymic masses. A normal thymus can be seen on CXR within 24 h after birth, after which it involutes and undergoes fatty infiltration within 2 years and is rarely seen after the age of 8 years. A normal thymus appears as a triangular sail towards the right of the mediastinum, without a local mass effect on vascular or airway structures [11].

Abnormalities can be seen as a focal or diffuse thickening of the anterior junction line and a loss of the retrosternal space on lateral views. Large anterior mediastinal masses can be evident by the presence of extra soft tissue density. The density of these soft tissue masses are similar to anterior mediastinal structures on radiography. Importantly, the loss or obscuration of normal anatomical borders may indicate thymic masses, by way of the ‘silhouette’ sign [12].

However, CXR has a poor sensitivity and specificity for thymic masses. Smaller thymic lesions are not radiographically apparent and it is impossible to distinguish between benign and malignant lesions with CXR. Invariably, cross-sectional imaging would be required for further characterisation and potential operative planning.

### 3.2. Computed Tomography and Magnetic Resonance Imaging

CT is currently the imaging modality of choice for the evaluation of mediastinal masses and surveillance. Its strengths include a capacity for high spatial resolution and the ability to characterise the lesion (location, morphology, shape, size, density, and enhancement) and its relationship to adjacent structures [13]. Herein, we briefly discuss the key imaging findings of benign and malignant anterior mediastinal lesions.

#### 3.2.1. Benign Lesions

##### Normal Thymus

A normal thymus on CT is located within the anterior mediastinum but may have various configurations and shapes—bilobed, inverted V, quadrilateral, or triangular [14]. On MRI, a normal thymus in young patients has a similar signal intensity to muscle on T1- and T2-weighted images [15]. As the gland involutes and fatty infiltration sets in with age, the signal becomes higher on both sequences [16].

##### Lipoma

Lipomas of the anterior mediastinum have been reported in 2% of primary mediastinal tumours [17]. Their appearance is avascular with homogeneous fat attenuation (−50 to −100 Hounsfield Units (HU)) and they have well-defined margins on CT [18]. Occasionally, the mass effect of these slow-growing tumours may cause local compression and symptoms. Malignant change is rare. These lesions appear hyperintense on T1- and T2-weighted MR images with decreased signal intensity on fat-suppressed images [17].

##### Thymic Hyperplasia

Thymic hyperplasia is characterised by an increase in the size and weight of the thymus gland with normal histology. This may occur within the setting of stress, corticosteroid therapy, radiation, chemotherapy, or surgery [19,20,21]. The two histological subtypes include true hyperplasia and lymphofollicular hyperplasia [22]. CT appearances show a symmetric diffuse enlargement of the gland which can retain its normal shape but also lose its bilobed appearance. Alternatively, it can also show up as diffuse soft tissue attenuation and may resemble a neoplasm [23]. The latter finding represents a deficit in the capabilities of CT in evaluating these thymic changes. Cross-sectional imaging with MRI may distinguish differences between thymic hyperplasia and tumours, such as microscopic or intravoxel fat which can occur in thymic hyperplasia but not in malignancy [16,23].

##### Thymolipoma

An uncommon benign encapsulated thymic tumour, thymolipoma is often discovered incidentally. It may be associated with chest pain, arrhythmias, cough, and dyspnoea within the setting of displacement of adjacent structures as they are typically large—weighing more than 500 g. Thymolipomas typically manifest on CT as large, pedunculated, well-defined anterior mediastinal masses containing 50–85% fat intermingled with areas of soft tissue attenuation [24]. These may conform to the shape of other mediastinal structures [25]. They may also lie within the thymus and mimic cardiomegaly or diaphragm elevation. On MRI, the fat component of the thymolipoma has a high signal intensity on both T1- and T2-weighted sequences and has a low signal on fat-suppressed imaging [15,25]. The latter is different from thymic tissue which has an intermediate signal intensity on all sequences.

##### Thymic Cysts

Hyperdense thymic cysts can be misinterpreted as solid lesions on CT and thus MRI can be used to differentiate thymic cysts from solid lesions. Generally thymic cysts are T1-hypointense, but the signal can vary depending on the cyst content as haemorrhage, fat or protein can increase the T1 signal [26]. All thymic cysts are T2-hyperintense [16]. A complete lack of enhancement on unenhanced and contrast-enhanced dynamic imaging proves the presence of a cyst. Congenital thymic cysts are unilocular with water intensity. Acquired cysts can arise with inflammation and are often multilocular [16,27,28]. An idiopathic multilocular cyst of the thymus is an acquired benign lesion which cannot be reliably distinguished from cystic components or malignant thymic lesions such as thymoma, Hodgkin’s lymphoma, and mediastinal germ cell tumours [29]. Generally, a serial six-month follow-up with MRI may be required to confirm stability or to assess for change [16].

##### Morgagni Hernia

Omental fat-containing hernia from the abdomen into the thorax through the foramen of Morgagni may frequently be mistaken for thymolipoma. These lie in a retrosternal or parasternal location, most frequently occurring on the right-hand side [30]. On CT they are reliably detected. They appear as a fat-containing mass in the lower anterior mediastinum. A discontinuation of the diaphragm and waist-like constriction of mesenteric folds (“Collar sign”) can also be present [31]. The key to diagnosis is the presence of a linear soft tissue opacity within the fat representing omental vessels, which can be traced down into the abdomen. MRI can help to characterise the hernial sac content; however, it is not the preferred imaging method for anticipated Morgagni hernia, particularly for acute and complicated cases given the relatively long duration and the sensitivity for motion artefacts. In cases of complicated hernias, a high signal on T1–T2 and fat-saturated sequences may represent ischaemic infarction.

##### Mediastinal Teratoma

The majority of mediastinal teratomas are mature teratomas that are histologically well defined and benign [32]. They are a rare entity with a low potential for malignant transformation. The location and composition of teratomas may help to differentiate between other anterior mediastinal masses. Mature teratomas may appear as well-defined lobulated masses protruding to one side of the thorax. Calcification in the form of bone elements or teeth may be seen in 26% of cases [7]. CT is able to reliably diagnose mature teratomas by evaluating the composition of the mass—fluid, soft tissue, and calcium and fat attenuations [15,33]. On MRI, teratomas typically demonstrate a heterogeneous signal intensity, which is reflective of the various combinations of elements. Fat–fluid levels within the lesion are diagnostic of a teratoma [34].

#### 3.2.2. Pre-Malignant and Malignant Lesions

An asymmetrical enlargement of the thymus on CT raises a concern for neoplastic processes. Thymic tumours are classified as epithelial (including thymoma and thymus carcinoma), lymphoma, thymolipoma, and germ cell tumours.

##### Thymoma

Thymoma is the most common neoplasm in the anterior mediastinum, accounting for 20% of mediastinal tumours [35]. It can be challenging to distinguish a thymoma from thymic hyperplasia, a normal thymus or other malignancy. Thymomas are typically closely related to the superior pericardium that is anterior to the aorta, pulmonary artery, and superior vena cava [15]. They also appear enhanced following contrast injection and measure roughly 5 to 10 centimetres [36]. Traditionally they are classified into 2 categories; invasive and non-invasive. Non-invasive thymomas usually appear encapsulated and are well defined with smooth contours and homogeneous attenuation, whereas an invasive thymoma is more likely to have lobulated or irregular contours, with areas of low attenuation and calcification [37]. Other useful signs include local invasion, distant metastases, and pleural or pericardial drop metastases [13]. Intravenous contrast is necessary for the staging of thymoma, as it may aid in evaluating vessel involvement [13]. On MRI, thymoma has an intermediate T1 signal intensity and an increased signal intensity on T2-weighted images [36]. Cystic areas may have a low signal intensity on T1 and a high signal intensity on T2-weighted images [38].

##### Thymic Carcinoma

Thymic carcinomas are rare and aggressive epithelial malignancies that frequently metastasise haematogenously to the lung, liver, brain, and bone. With CT, they are often seen as large, ill-defined, and heterogeneous soft tissue masses [15,39]. Occasionally, areas of haemorrhage, necrosis, and calcifications are seen with associated pleural and pericardial effusions. High-risk imaging features, such as local invasion and lymphadenopathy, should prompt an imaging evaluation for distant metastases [40]. Intravenous contrast is required to visualise direct vascular involvement as well as in aiding the identification of metastatic disease. On MRI, thymic carcinomas are typically identified by an intermediate signal on T1 and a high signal on T2-weighted imaging [15,41]. However, a pitfall of MRI in assessing invasion is its inadequacy in evaluating lung parenchyma due to the presence of air. A high standardised uptake value (SUV) in Fluorodeoxyglucose (FDG)-PET may be useful in differentiating thymic carcinoma from thymic neoplasms, hyperplasia, or normal physiological uptake [42]. Up to 65% of the cases of initial thymic carcinomas are estimated to have metastasised at the time of diagnosis, with common regions including the regional thoracopleural nodes, lung, liver, and brain [15].

##### Lymphoma

Involvement of the thymus in Hodgkin’s lymphoma is common, with roughly a third of patients with a new diagnosis presenting with an enlarged thymus. This may be in the context of primary involvement or due to an infiltration from adjacent lymph nodes. On CT, the thymus may be diffusely enlarged or have a multinodular appearance in thymic lymphoma [21]. This is associated with mediastinal lymphadenopathy. Mediastinal MRI can help to differentiate a lymphoma from a thymoma as well as assess for pericardial involvement [41]. Involved nodes have a homogeneous high signal intensity greater than muscle but similar to fat on T2-weighted imaging [41]. Hodgkin’s lesions may appear heterogeneous with mixed areas of high and low signal intensity on T2 sequences, which may be reflective of nodular sclerosing Hodgkin’s disease, as histologically there are fibrotic areas interspersed with cellular areas [15].

##### Germ Cell Tumours

Non-teratomatous germ cell tumours (NTGCT) are rare, rapidly growing, and malignant tumours that frequently affect the anterosuperior mediastinum [43]. They are subdivided into seminomatous and non-seminomatous tumours. They appear as bulky, ill-circumscribed, and lobulated masses. Primary mediastinal seminomas almost exclusively occur in males during their second to fourth decade of life. They typically appear to be homogeneous on CT and show minimal enhancement after administrating contrast. There may be areas of degeneration within the setting of haemorrhage and necrosis [43]. Furthermore, there may be evidence of metastases to the lymph nodes and bone. Non-seminomatous tumours present as large masses with marked heterogeneous attenuation on CT with areas of low attenuation which may represent necrosis. They may be associated with local invasion, distant metastases to regional nodes, or lung, pleural, and pericardial effusions. On MRI, these tumours typically show internal heterogeneous intensities with areas of high signal likely reflecting degenerative cystic changes on T2-weighted imaging [15].

##### Thymic Neuroendocrine Tumours

Primary neuroendocrine tumours (NET) of the thymus, also known as carcinoid tumours, are exceedingly rare yet aggressive cancers that comprise less than 5% of anterior mediastinal masses [15]. These tumours appear similar to thymomas on CT and MRI; however, they usually present at a much more advanced stage with other sequelae including superior vena cava obstruction, a finding that is uncommon with thymomas [15]. Additionally, these tumours may be associated with paraneoplastic carcinoid syndrome [15]. On CT and MRI, they present as lobulated lesions of the thymus with central necrosis or haemorrhage, often with local invasion and the potential to metastasise to regions including bone [15]. The diagnosis of a thymic NET requires multimodal work-up, often with PET imaging, somatostatin receptor-based diagnostic imaging modalities and/or neuroendocrine biochemical markers.

##### Neurogenic Tumours

Neurogenic tumours represent around 20% of mediastinal tumours in adults and up to 35% in paediatric populations. Peripheral nerve sheath tumours such as schwannomas are the most common of these tumours and appear as convex, dumbbell-shaped lesions with or without cystic change, calcification, or haemorrhage on CT. On MRI they appear similar, but they may have a heterogeneous high signal intensity on T2-weighted imaging [24]. Neurofibromas are non-encapsulated peripheral nerve tumours which also appear similar to schwannomas; however, they are less likely to exhibit cystic or haemorrhagic change. Follow-up imaging of neurofibromas typically shows robust stability; however, a sudden evolution, such as an increase in size on repeat CT or MRI, may suggest a transformation to a malignant peripheral nerve sheath tumour [24]. Additional features of malignant transformation include a contrast enhancement on MR and a heterogeneous signal.

Sympathetic ganglion tumours represent 25% of neurogenic mediastinal tumours and arise from neurons rather than the peripheral nerve sheath [24]. Ganglioneuromas are benign manifestations of these tumours and appear as ‘whorled’ curvilinear bands with a hypointense signal on T2-weighted MR [24]. On CT, they may be well-defined or ill-defined masses which orient themselves on the anterolateral surface of vertebrae. Conversely, neuroblastomas are aggressive tumours that exhibit heterogeneity, haemorrhage, necrosis, cystic change, or calcification, often with distant metastases at the time of imaging [24].

Paragangliomas are rare, with two key types including aortopulmonary paraganglioma and aortosympathetic paraganglioma. Both are hypervascular tumours that enhance brightly on contrast CT. On MRI, they demonstrate curvilinear and punctate signal voids due to the high-velocity flow of the tumour blood supply, a phenomenon colloquially described as ‘salt and pepper’ in morphology [24].

### 3.3. Other Imaging Modalities

CEUS is a method which examines blood flow, and is another imaging modality used in the evaluation of thymic lesions [44]. CEUS has been found to improve the diagnostic accuracy in biopsies of anterior mediastinum lesions compared to regular ultrasound due to varying enhancement pattern, distribution and time [45]. Although the literature has described specific features of thymomas, thymic carcinomas and lymphomas analysed by CEUS, in the context of an evidence-based evaluation, there is little evidence to suggest that CEUS can differentiate between benign and malignant thymic lesions on imaging alone, without the biopsy [46,47].

Endobronchial ultrasound (EBUS) is a further method of diagnosis of anterior mediastinal lesions [48]. Similar to CEUS, EBUS is often performed in conjunction with biopsy [49]. It is minimally invasive and has been shown to be accurate in the diagnosis of anterior mediastinal lesions when a non-invasive diagnostic approach, such as with cross-sectional imaging, was inconclusive [49]. EBUS as an imaging modality without the use of biopsy is unlikely to provide significant evidence to distinguish between types of thymic lesions.

## 4. Insights into First-Line Magnetic Resonance Imaging in the Evaluation of the Thymic Lesion

Overall, CT has been found to be equal, if not superior, to MRI in the diagnostic accuracy for anterior mediastinal tumours, except in the case of the thymic cyst. Assessment of diagnostic accuracies of these two modalities suggests an overall average correct first-choice diagnosis of 61% for CT and 56% for MRI [50]. These rates vary depending on pathology, with thymic cysts identified with an accuracy of 71% for MRI and 46% for CT and similar accuracy rates for thymoma (CT: 83%, MRI: 84%). However, evidence from the literature suggests that MRI would be advantageous in distinguishing between solid and cystic anterior mediastinal tumours in settings where CT is equivocal [50]. MRI is also a good alternative to CT if exposure to radiation is a concern, such as in younger patient or pregnant populations, or if there is a contrast allergy or poor renal function. Despite this, clinicians face diagnostic difficulties with lesions of the thymus and anterior mediastinum, with recent observational evidence reporting unnecessarily high rates of non-therapeutic thymectomy [4,5,51]. Reasons include difficulties with the interpretation of thoracic CT imaging for the heterogeneous pathologies of the anterior mediastinum [51]. A confounding factor is the varied protocols for thoracic CT imaging. Specifically, Ackman et al. report in an observational study of unnecessary non-therapeutic thymectomies, that 19% of patients had thoracic CT imaging without contrast, with the remainder having 35-second delayed contrast thoracic CT [51]. Their study demonstrates the challenges in discerning the lesional differences of anterior mediastinal and thymic lesions on CT imaging. Other studies suggest assessing pre- and post-contrast attenuation as a more sensitive approach to the assessment of the thymic lesion [52]. However, it appears that such methods may only be valid for certain age ranges [52]. The question then becomes whether upfront MRI, rather than CT, is able to be utilised practically, safely, and with lower risk profiles than thymectomy.

Current guidelines continue to advocate for CT as the initial test for the patient with a suspected or incidental anterior mediastinal or thymic lesion [3,8,10,53]. MRI is considered an adjunct to CT in the further evaluation of these lesions given its superior resolution for soft tissue, adopting a second or third-line place in the diagnostic algorithm [54]. Currently, there is lack of evidence characterising the sensitivity of MRI for the evaluation of these lesions as a standalone upfront modality. Additionally, there is a lack of robust real-world evidence to suggest that MRI evaluation of such lesions reduces the rate of non-therapeutic thymectomy when compared to CT. Nonetheless, MRI has specific benefits over thoracic CT. MRI has a higher capacity to resolve intricate details to better elucidate differences between cystic lesions with regards to complexity such as the presence of solid components, necrosis, septa, and local invasion [55]. Furthermore, MRI has the ability to detect intravoxel and microscopic fat, thereby allowing a greater capability in distinguishing thymic hyperplasia from thymoma [56]. However, interobserver variability and therefore expertise are the key barriers to an accurate diagnosis, with studies that highlight significant variability in the accuracy of MRI compared to CT diagnosis based on pathology [50].

The use of MRI as an upfront test for evaluation at the point of diagnosis has been proposed as a key method to reduce non-therapeutic thymectomy. Understandably, the opportunity for a better non-invasive and non-surgical test such as MRI to evaluate these lesions compared to thymectomy is highly appealing, both from a resource perspective for healthcare systems as well as a risk perspective for patients. However, the diagnostic utility of MRI has yet to be clarified for this indication. There have been arguments that suggest dual modality imaging, such as with index thoracic CT and MRI, may be an avenue to explore to reduce misinterpretation, misdiagnosis, and/or thymectomy [52]. However, this approach is challenged by the practicality of resource allocation and cost, particularly in settings where MRI is not readily available.

Significant barriers remain for integrating MRI as an upfront modality for the evaluation of thymic lesions. Central to this are discussions surrounding its accessibility and financial practicality. In Australia, the cost is greater than that of CT due to the referral pathways and the need for specialty involvement [57]. This occurs because of background access issues due to the scarcity of MRI in relative proportion to CT and a greater cost in operating MRI technologies [58,59,60]. However, it has been estimated that in addition to surgical risk and morbidity, the inflation-adjusted cost of a non-therapeutic thymectomy is equivalent to USD11,072 [61]. Arguably, MRI as an alternative may seem more appealing in light of the surgical expenditure and balance of risk. An additional argument considers the cost of MRI compared to CT, with MRI estimated to incur a cost 1.5 to 2 times greater than CT [16]. This may be related to the machinery itself, alongside the greater demand for highly skilled technicians, radiologists, and supporting staff [60,62]. Furthermore, with the diagnostic complexities of anterior mediastinal lesions, the need for further subspecialist radiologists and technicians adds further barriers to widely accessible thoracic MRI for these indications. These issues become more pronounced in regions challenged by access to medical technologies, such as lower to middle income countries as well as rural and remote regions [60]. Specifically, in jurisdictions or services where MRI is not available, it may be impractical to arrange the transfer of a patient for MRI for further characterisation when CT or even surgical thymectomy are local options. In adopting a patient-centred approach, clinicians must also consider the practicality of MRI, CT, or surgery based on relevant expectations from the patient. In particular, for those from resource-poor or rural/remote settings, connections to family, financial difficulty, and local work priorities are additional barriers to referring such patients for MRI at distant centres. In these circumstances, the potential for improved accuracy with MRI may not be justifiable and therefore CT or thymectomy may be more appropriate. Patients should be counselled regarding these potential options, including the impact of cost, time of each pathway, and quality of life, with the latter particularly related to surgical approaches, to allow for well-informed and shared decision making. Further research and validation of optimised referral pathways, to stratify those who would benefit from MRI and those who would not would provide more streamlined decision making for clinicians who are challenged with these diagnostic dilemmas.

## 5. Conclusions

Challenges continue to exist in working up the patient with anterior mediastinal or thymic lesions. Current guidelines continue to support the role of thoracic CT imaging in the initial work-up of patients with these lesions with high rates of non-therapeutic thymectomies still encountered. Despite this, the rates of non-therapeutic thymectomy continue to remain unacceptably high, with downstream challenges to the responsible use of healthcare resources as well as the surgical risk to the patient. Upfront MRI is becoming increasingly considered as a potential method of higher sensitivity evaluation to reduce non-therapeutic thymectomy; however, its role in the evaluation of the anterior mediastinum remains poorly characterised. There is a need for larger prospective trials alongside cross-discipline collaboration to evaluate the efficacy, practicality, accessibility and cost of upfront MRI before it can be considered as a primary diagnostic tool to evaluate the anterior mediastinum.

## Data Availability

Data utilised for the generation of this manuscript were obtained from publicly available peer-reviewed articles. No manipulation or generation of new data occurred in the research process.
